# Bio-Inspired Flexible-Wall Squeezing Mixer with ALE-CFD-Based Actuation Optimization and Fluorescence-Imaging Assessment of Outlet Mixing Uniformity

**DOI:** 10.3390/biomimetics11040284

**Published:** 2026-04-20

**Authors:** Wen Yuan, Zhihong Zhang

**Affiliations:** Faculty of Intelligence Technology, Shanghai Institute of Technology, Shanghai 201418, China; 236101138@mail.sit.edu.cn

**Keywords:** bio-inspired laminar mixer, flexible-wall actuation, deformation-driven mixing, two-dimensional ALE-CFD, response surface methodology, fluorescence-based outlet homogeneity, trend-level experimental corroboration

## Abstract

Efficient mixing is a persistent bottleneck in agricultural and agrochemical processing, where rapid and uniform mixing must be achieved under laminar flow with low energy input and gentle shear. Inspired by peristaltic transport in biological systems, this study investigates a bio-inspired flexible-wall squeezing mixer and establishes a two-dimensional computational framework to quantify how periodic wall deformation governs scalar homogenization in a flexible conduit. An Arbitrary Lagrangian–Eulerian dynamic mesh approach is implemented to resolve moving boundaries and to prescribe actuation, enabling the systematic evaluation of the separate and coupled effects of peak wall-normal velocity amplitude *A* and actuation frequency *f* on mixing performance. Mixing effectiveness is quantified using a variance-based mixing index MI and a sustained-threshold mixing time ts, and response surface methodology is employed to map the A–f design space and interpret the roles of time-dependent shear, interfacial stretching and folding, and vortex intensification. Relative to a non-actuated baseline, a peak wall-normal velocity amplitude of 3 × 10^−3^ m s^−1^ at 2 Hz reduces *t*_s_ by 21.3%. At fixed *f* = 3 Hz, increasing *A* from 1 × 10^−3^ to 4 × 10^−3^ m s^−1^ shortens *t*_s_ by 10.2%, while at fixed *A* = 3 × 10^−3^ m s^−1^, raising f from 1 to 5 Hz further decreases *t*_s_ by 6.6% with diminishing gains at the lowest frequencies. The response surface identifies an operating optimum at *A* = 4 × 10^−3^ m s^−1^ and *f* = 5 Hz, achieving a peak MI of 0.9557 and a minimum *t*_s_ of 7.81 s. A periodically squeezed physical mixing loop was further examined using fluorescence imaging to assess outlet homogeneity trends. The stabilized outlet coefficient of variation (CV) decreased from about 0.65 without squeezing to 0.60 at 1 Hz and 10 mm s^−1^, 0.58 at 2 Hz and 10 mm s^−1^, and 0.54 at 2 Hz and 30 mm s^−1^, indicating that stronger and faster actuation improves outlet uniformity. The numerical and experimental results are therefore interpreted jointly as mechanistic and trend-level evidence, while a rigorous quantitative prediction for the cylindrical compliant device will require future three-dimensional, compliance-resolved simulations and broader experimental benchmarking.

## 1. Introduction

In modern agriculture, mixing serves as a critical unit operation that enhances input efficiency and optimizes field outcomes. By employing inline mixing—wherein concentrates, adjuvants, and carriers are precisely metered and blended in real time—growers can implement variable-rate applications for spraying, fertigation, and seed treatment directly based on prescription maps [1,2,3,4,5]. Homogenization is often the critical determinant of a process’s desirable quality. Mixing behavior differs radically between dissolving, suspending, and aerating phases, driven by system-specific transport phenomena [6]. Liquid-phase mixing is particularly complex, requiring a balance of large-scale convection, turbulent eddy transport, and molecular-scale dispersion to achieve the expected uniformity. Consequently, a central challenge in process engineering is the development of effective strategies to optimize mixing kinetics and dynamics, which is profoundly influenced by a range of physical and chemical parameters [7].

Conventional mixing technologies predominantly rely on rigid mixer architectures and are commonly divided into static and dynamic types. Static mixers employ fixed internal elements, such as helices, corrugated inserts, or perforated plates, that reorient the flow and create velocity gradients, passively enhancing mixing by the repeated splitting, folding, and lamination of fluid streams [8]. Dynamic mixers, in contrast, use actively moving components (e.g., impellers or turbines) to impose forced convection and, when applicable, turbulence within the vessel [9]. Generally, both classes pursue the same objective to maximize dispersion by stretching, splitting, repositioning, and recombining fluid elements, often aided by vortex generation and chaotic advection.

From a practical standpoint, static mixers offer no moving parts, compact design, and low maintenance. However, their operation incurs an additional pressure drop that requires pumping power, and performance can deteriorate in highly viscous fluids or at very low Reynolds numbers, where stagnant zones and fouling are more likely to develop [10,11,12,13]. Dynamic mixers provide the precise tunability of mixing intensity via rotational speed and impeller geometry, enabling broad control over energy dissipation and residence time distribution; yet they typically demand a higher specific energy input, entail seals and bearings with mechanical complexities and may damage shear-sensitive materials such as emulsions or long-chain polymers [14,15,16].

These trade-offs between controllability and energy demand on the one hand, and simplicity and pressure-drop penalties on the other, motivate alternative strategies that actively perturb laminar flows without internal rotating parts. In this context, bio-inspired mixers with deformable boundaries introduce periodic disturbances to promote interfacial renewal and mass transfer, providing the conceptual bridge to the flexible, peristalsis-driven approach investigated in this work. This class of mixers has been extensively investigated through both experimental and numerical studies. For instance, Liu et al. [17] conducted combined numerical and experimental work to measure the pressure drop in SMX static mixers. Regner et al. [18] characterized static mixer performance using metrics such as the Z-factor, vorticity, and striation thinning rate, finding that vortices in Kenics KM and Lightnin mixers enhanced material deformation and that mixing was more efficient at lower flow rates where obstructive separation zones are less likely to form. Research has also focused on the influence of parameters like flow velocity, pipe diameter, and the geometry and number of blade elements on mixing performance. Parvizi et al. [19] explored multiple factors impacting the efficiency of dynamic mixers, concluding that axial impellers are superior to radial ones in terms of energy efficiency and that blade geometry significantly affects flow patterns and shear stress distribution. Wang et al. [20] employed computational fluid dynamics (CFD) to evaluate the flow patterns and mixing of highly viscous fluids, demonstrating that extensional flow is more effective than shear flow for achieving uniform concentration distributions. These findings position peristalsis-driven, deformable-boundary mixers as a promising low-complexity pathway to controllable, energy-efficient laminar mixing with reduced pressure-drop penalties.

A mixer design requires a deep understanding of fluid mixing dynamics. It facilitates rapid processing and ensures uniform product quality. While macroscopic phenomena such as mixing time and flow patterns have been the traditional focus, biological-derived concepts are gradually reshaping traditional frameworks. Drawing inspiration from the efficiency of biological systems [21], the concept of bionics has inspired novel system performances, especially flexible and biomimetic mixers [22]. It is characterized by its use of dynamically deforming walls to lead internal mixing processes. Unlike typical rigid structures, they employ boundaries to generate controlled fluid motion through periodic squeezing, peristaltic motions, or other biomimetic deformations. This remains an emerging field in industrial applications, where research, although still limited, shows promising growth. This field’s potential lies in replicating biological systems’ energy management and adaptative response, providing more sustainable and scalable solutions than traditional methods. A prime biological prototype is the stomach organ, which employs powerful peristaltic contractions to effectively mix digestive fluids and chyme, by means of a combination of traveling waves and compartmentalized segmentation [23]. Several laboratory tests have been carried out to represent the digestive process. Building on this line of research, Liu et al. [24] showed experimentally that integrating soft-elastic baffles into a soft-elastic mixer markedly enhances Newtonian mixing; the mixing time decreases monotonically as the number of baffles increases, with a single soft-elastic baffle already cutting mixing time by 34% at 1.0 Hz. Delaplace et al. [25] experimentally established mixing time correlations for soft-wall mixers driven by wall vibrations; Zhuang et al. [26] demonstrated an origami-based bionic mixer that improves the blending of high-viscosity media; Hernalsteens et al. [27] built and evaluated a soft-elastic tubular mixer, characterizing its aeration/mixing/temperature-control performance; and Zhang et al. [28] systematically showed that symmetric peristalsis and optimized wall structures intensify mixing. However, investigating these mechanisms is still challenging due to difficulties in visualizing and quantifying complex internal flows.

Computational fluid dynamics can be useful to evaluate these mixers by enabling the detailed analysis of fluid–structure interactions that are otherwise almost inaccessible experimentally [29,30,31,32,33]. It overcomes the material and experimental limitations inherent in studying traditional mixers, also vastly improving the predictability and controllability of the mixing process [34]. High-fidelity simulations provide a solid foundation for achieving highly efficient industrial operations and consistently superior product quality. The walls of a flexible mixer, composed of soft, elastic materials, actively participate in the mixing process. Their deformation dynamically alters the internal flow domain, enhancing fluid dynamic instabilities and promoting mass transfer between different components. Ferrua and Singh [35] modeled antral contractions and estimated how the viscosity reduces mixing jets and increases the internal pressure. Building on this idea, Zou et al. [36] simulated peristalsis and segmentation movements, predicting that they could enhance mixing by improving fluid convection and flow control. Similarly, Li et al. [37] investigated boundary-driven mixing in soft-elastic mixers with 2D-CFD models, identifying key geometric parameters, such as aspect ratio, and baffle positioning, that govern mixing homogeneity.

This study moves beyond a single-case demonstration by establishing a design-oriented actuation–performance framework for laminar inline mixing under periodic flexible-wall squeezing. Actuation is parameterized by the peak wall-normal velocity amplitude A and frequency f within an ALE moving-mesh CFD formulation, and mixing performance is quantified using complementary variance-based measures, including a sustained-threshold mixing time. Trends are summarized through an operating map and a response surface surrogate model with reported fit indicators. A fluorescence-imaging experiment based on CV measurements is further used as a proof-of-concept and trend-level corroboration platform to examine whether the same ordering of mixing performance is observed in the physical device. Motivated by a bio-inspired mixing concept, this study starts with a virtual prototype, a flexible-walled conduit in which periodic wall squeezing perturbs laminar flow. Because direct, in situ visualization of internal flow structures in soft-walled small-scale mixers is inherently challenging, two-dimensional numerical simulations were conducted to visualize the internal dynamics and to efficiently screen actuation parameters for subsequent design iterations and optimization. The simulations were configured within an ALE dynamic mesh framework to reproduce the theoretical influence of prescribed wall-motion frequency and amplitude on transport and mixing. This computational approach links mixing index and mixing time device-level metrics to underlying mechanisms, including vortex strengthening, interfacial stretching, and shear-induced dispersion, thereby providing technical references for geometry and actuation choices in future physical implementations. Accordingly, the study pursues three objectives tied directly to the visualization and design-optimization challenge: (1) implement a high-fidelity ALE dynamic mesh model of periodic wall squeezing; (2) systematically evaluate the impact of squeezing actuation on mixing effectiveness using quantitative metrics; and (3) quantify the separate and joint effects of wall-motion frequency and amplitude, investigating the operating conditions for performance to inform the future design of bio-inspired flexible mixers. Prior studies have shown that deformable-boundary and soft-elastic mixers can enhance laminar mixing, yet two issues remain insufficiently addressed for design-oriented use. First, the separate and coupled effects of actuation amplitude and frequency have rarely been mapped systematically within a compact operating framework. Second, available physical experiments are typically device-specific, whereas rapid parameter screening still requires simplified numerical models that capture the dominant transport mechanism without claiming full geometric or structural fidelity. Accordingly, the present work establishes a two-dimensional ALE-based kinematic model for an actuation-oriented trend analysis of a flexible-wall squeezing mixer. The numerical framework is used to identify how prescribed wall-motion amplitude and frequency affect scalar homogenization, mixing time, and operating-region performance. A bench-scale fluorescence-imaging experiment is further used as a proof-of-concept and trend-level corroboration platform to examine whether the same ordering of mixing performance is observed in the physical device. The objective is therefore mechanistic interpretation and parametric design guidance, rather than one-to-one quantitative reconstruction of the three-dimensional compliant cylindrical prototype.

## 2. Materials and Methods

### 2.1. Geometry and Computational Domain

**Figure 1:**
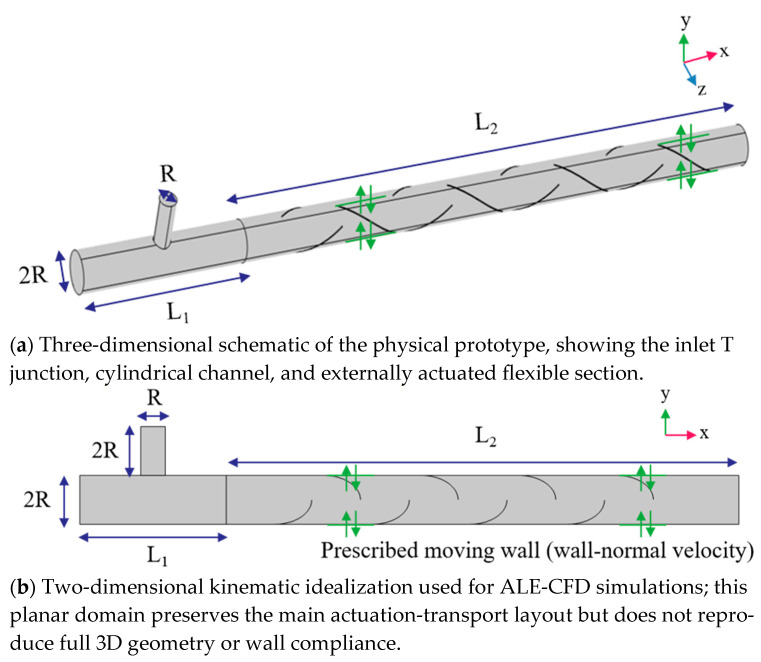
Physical prototype and corresponding two-dimensional kinematic idealization used for CFD trend analysis.

The computational geometry idealized an experimentally constructed mixer (Figure 1) while preserving the flow-relevant features. The device comprised two sections: a rigid T-junction that served as the inlet manifold and established two initially segregated streams, and a flexible-wall squeezing section in which mixing was evaluated. The dimensions are defined by three parameters. The T-junction had a primary channel of length *L*_1_ and inner diameter 2*R*, with a perpendicular secondary inlet (Inlet 2) of inner diameter *R*. Downstream, the flexible mixer had total length *L*_2_ and maintained a uniform inner diameter 2*R* from its entrance to the outlet. Here, *R* was used as a reference length scale.

In the corresponding physical prototype developed by Liu et al. [38], an external motor imposed periodic squeezing on the flexible section, thereby driving time-varying wall deformation that directly affected the internal flow and mixing. In the simulations, this actuation was presented by prescribing the wall motion via an ALE dynamic mesh, allowing a direct link between actuation parameters and homogenization metrics. The T-junction was treated as hydraulically rigid, and the subsequent analysis focused on the flexible downstream section where the effects of deformation on scalar transport were most pronounced. To establish a clear correspondence between the physical prototype and the numerical model, a two-dimensional computational domain was constructed as an effective representation of the in-plane flow within the mixer. This two-dimensional kinematic projection was intended to capture the dominant deformation-driven transport mechanism responsible for interface stretching and folding under periodic squeezing, while deliberately excluding secondary three-dimensional effects such as azimuthal recirculation, axial variations associated with the helical geometry, and end-wall-induced flows. Accordingly, the present model was used to establish mechanistic trends and operating region guidance as functions of A and *f*, rather than to reproduce all geometric details of the physical prototype. The limitations of this idealization and the need for future three-dimensional, compliance resolved modeling are stated explicitly to prevent over-interpretation of the quantitative magnitudes.

This model does not correspond to a literal planar cross-section of the three-dimensional device; instead, the essential kinematic features of the three-dimensional helical wall deformation are projected onto a two-dimensional plane, where the periodic wall motion is represented by a set of time-dependent moving boundaries. Under laminar operating conditions, the flow is assumed to be symmetric and free of azimuthal variations. In this idealized framework, the axial cross-section of the physical device is mapped onto a planar channel of height 2*R*, thereby preserving the hydraulic diameter, the cross-sectional area per unit depth, and the characteristic length scales that govern convection and diffusion. Where simplified geometry elements are introduced in the 2D domain, they are used as surrogate curves to represent the projected kinematic footprint of the three-dimensional helical deformation and any secondary geometric details in a hydraulically consistent manner. This surrogate representation is intended to preserve the first-order confinement and streamline deflection while keeping the boundary-driven deformation mechanism unchanged. Under the present low-Reynolds-number laminar conditions, the mixing enhancement is dominated by unsteady vortical advection and interface stretching induced by the prescribed periodic wall motion, so the use of smooth surrogate curves is a reasonable abstraction for capturing mechanism and parametric trends in the amplitude–frequency operating space. Consistent with this intent, the accompanying fluorescence experiment is used to validate these trends at the qualitative level. This simplified two-dimensional formulation retains the dominant deformation-driven transport mechanism induced by periodic wall motion, which is responsible for the primary stretching and folding of fluid elements. Meanwhile, secondary three-dimensional effects are intentionally neglected, including azimuthal recirculation, axial variations associated with the helical geometry, and end-wall-induced flows.

Although the physical prototype is three-dimensional and cylindrical, the present two-dimensional abstraction is designed to preserve the first-order transport scales that govern advection and molecular diffusion under the investigated laminar conditions. In particular, by maintaining the effective confinement and hydraulic length scales per unit depth, the model captures the cross-stream deformation imposed by periodic wall motion and the associated renewal of scalar gradients that drives homogenization. Potential three-dimensional contributions, including azimuthal secondary motions, axial variations associated with helical deformation, and end-wall-induced recirculation, are not resolved in the current framework and may influence quantitative values in a fully resolved device. However, within the low-Reynolds-number regime targeted here, these effects are expected to be secondary relative to the boundary-driven, time-dependent shear and interface stretching generated directly by the imposed squeezing kinematics. The present CFD framework should therefore be interpreted as a two-dimensional kinematic idealization designed to resolve the dominant deformation-driven transport mechanism and to identify parametric trends in the actuation space, rather than as a fully resolved quantitative replica of the three-dimensional helical prototype.

### 2.2. Multiphysics and Species Transport Model

A coupled Multiphysics framework was formulated to quantify how the periodic squeezing of a flexible wall effects scalar homogenization in the proposed mixer. The model integrated unsteady hydrodynamics, dilute-species transport, and an ALE description of wall-induced mesh motion. The fluid domain was treated as single phase, water served as the continuous Newtonian liquid, and ethanol, introduced at trace concentration, acted as a dilute marker species.

The hydrodynamics were solved for an incompressible, isothermal liquid with constant density and viscosity equal to those of water over the examined concentration range. Ethanol was modeled as a passive scalar governed solely by advection and diffusion, with constant diffusivity unless otherwise stated. Accordingly, the coupling was one way, from flow to species, so that the scalar field did not feedback on the velocity, pressure, or material properties. Phase change, interfacial phenomena, chemical reactions, and gravitational effects were neglected.

The periodic deformation of the flexible section was prescribed through ALE kinematics, which updated the computational mesh each time step while enforcing no-slip on the moving wall. Dirichlet conditions were imposed at the inlets for velocity and ethanol concentration. A convective, stress-free boundary condition was applied at the outlet to minimize artificial reflections, and zero normal species flux was enforced on all solid boundaries.

Under these assumptions, the governing Equations (1) and (2) comprised the continuity and incompressible Navier–Stokes momentum equations for the liquid phase, together with the transient advection–diffusion equation for ethanol. The flow and scalar fields were advanced in a tightly coupled manner consistent with the one-way coupling hypothesis, and all results reported herein reflect the stated constitutive and boundary idealizations.

(1) ρ∇⋅u=0

(2) $$\rho \frac{\partial u}{\partial t}+\rho (u\cdot \nabla )u=\nabla \cdot [-pI+K]+F$$

where *t* is the time (s), *ρ* is the fluid density (kg m^−3^), *u* is the fluid velocity vector (m s^−1^), *p* is the pressure (Pa), *I* is the identity tensor (dimensionless), *K* is the viscous stress tensor (Pa), and F represents external volume forces (N m^−3^).

The mass transport of ethanol (solute) in water (solvent) was governed by the convection–diffusion equation for each chemical species *i* (Equation (3)):

(3) $$\frac{\partial c_{i}}{\partial t}+\nabla \cdot J_{i}+u\cdot \nabla c_{i}=R_{i}$$

where c_i_ is the concentration (mol m^−3^), *R_i_* expresses the production/consumption of *i* (mol m^−3^ s^−1^), and *J_i_* is the diffusive flux according to Equation (4) (Fick’s Law):

(4) $$J_{i}=-D_{i}\nabla c_{i}$$

where *D_i_* is the diffusion coefficient, with 1.22 × 10^−9^ m^2^/s fixed for ethanol in water at 25 °C. This equation balances transient changes *(∂c_i_/∂t*), diffusion (∇⋅*Ji*), advection (*u*⋅∇*c_i_*), and reactions (*Ri*).

Ethanol was treated as a passive scalar transported by advection and molecular diffusion with constant diffusivity. The molecular diffusivity was fixed at *D*_i_ = 1.22 × 10^−9^ m^2^/s, corresponding to the mutual diffusivity of ethanol in water at 25 °C under near-infinite-dilution conditions, which is consistent with the dilute-tracer assumption adopted in this study. In all simulations, the working fluid was treated as an incompressible Newtonian liquid with constant properties representative of water at 25 °C. Specifically, the density and dynamic viscosity were set to *ρ* = 997 kg m^−3^ and *μ* = 0.89 mPa s, respectively. To characterize the flow regime, the Reynolds number was defined as *Re* = ρ U *D*_h_/*μ*, where U is the bulk velocity and Dh is the hydraulic diameter of the corresponding section (*D*_h_ = 2R for the main channel and Dh = R for the secondary inlet in the idealized 2D mapping). The resulting Reynolds numbers based on the inlet bulk velocities reported in Table 1 are provided explicitly in Table 1 to ensure consistent reporting of the operating regime and to support the laminar-flow modeling assumption used throughout this work.

In the simulations, ethanol was used as a representative passive scalar because its mutual diffusivity in water is well documented and can be prescribed consistently in the advection–diffusion formulation. In the experiments, a dilute fluorescent aqueous tracer was used to enable the optical quantification of outlet homogeneity via fluorescence imaging. Although the tracers were different, both correspond to dilute tracers transported in an aqueous carrier under laminar conditions, and therefore did not materially alter the bulk rheology or the flow kinematics. In the present operating range, transport was strongly advection dominated, with Péclet numbers on the order of 10^6, so the mixing enhancement was governed primarily by wall-driven deformation, unsteady advection, and interface stretching and folding. Consequently, differences in molecular diffusivity were expected to affect absolute homogenization rates but not the monotonic trends and relative ranking of mixing performance across actuation conditions, which is the basis of the trend-level CFD–experiment comparison adopted in this work.

### 2.3. ALE-Based Dynamic Meshing and Wall-Actuation Model

Periodic squeezing was imposed using an ALE dynamic mesh on a two-dimensional hybrid unstructured grid comprising a triangular core and wall-aligned quadrilateral inflation layers along all solid and actuated boundaries. Near-wall resolution targeted y+ ≤ 1 at the peak-cycle Reynolds number using a first-layer height of 1.0 × 10^−4^ m, 12 prism layers with a growth ratio of 1.15, and a total inflation thickness of 1.8 × 10^−3^ m. The minimum cell size equaled the first-layer height, and the core edge length ranged from 5 × 10^−4^ to 1 × 10^−3^ m. A mesh independence study (9377; 13,249; 19,010; 32,007 elements) supported the use of the 1.901 × 10^4^ element grid for all reported simulations. The ALE strategy has been well suited to problems with moving boundaries and transport across deforming interfaces [35,39] because it allowed the computational mesh to follow wall motion while maintaining fidelity to the underlying flow physics.

In the presented implementation, wall actuation was prescribed kinematically and enforced through the mesh velocity at boundary nodes, while the no-slip condition ensured that the fluid velocity matched the instantaneous wall velocity. Interior nodes were displaced by diffusion-based smoothing to preserve element quality and avoid excessive skewness as the walls deformed. This formulation combines the advantages of Lagrangian and Eulerian descriptions by permitting boundary-conforming motion without advecting the mesh with the bulk flow.

Symmetric, time-periodic squeezing of the flexible section was imposed by specifying equal-magnitude, opposite-sign wall-normal velocities on the upper and lower walls. Using *A* (m s^−1^) for the velocity amplitude and *f* (Hz) for the actuation frequency, the prescribed boundary velocities were defined as (Equations (5) and (6)):

(5) $$V_{\mathrm{up}}(t)=A\sin(2\pi ft)$$

(6) $$V_{\mathrm{down}}(t)=-A\sin(2\pi ft)$$

where *A* represents the velocity amplitude of the squeezing motion (m s^−1^), and *f* is the squeezing frequency (Hz).

Therefore, the walls move 180° out of phase and periodically constrict and relax the channel. This choice provides a zero-mean, purely oscillatory actuation that isolates deformation-driven transport from any imposed net throughput. Boundary conditions followed Section 2.2, velocity-inlet conditions were applied at both inlets with uniform (“top-hat”) profiles of magnitude *u*_1_ and *u*_2_, respectively, together with the specified ethanol concentration to enforce complete initial segregation of the two streams at their entry planes. The outlet employed a convective, stress-free condition, and all solid boundaries were impermeable with zero normal species flux. No pre-mixing manifold, interfacial mass transfer, phase change, or reactions were considered, ensuring that any downstream homogenization arose from the computed unsteady flow and diffusion within the actuated mixer.

### 2.4. Grid Independence Study

**Figure 2:**
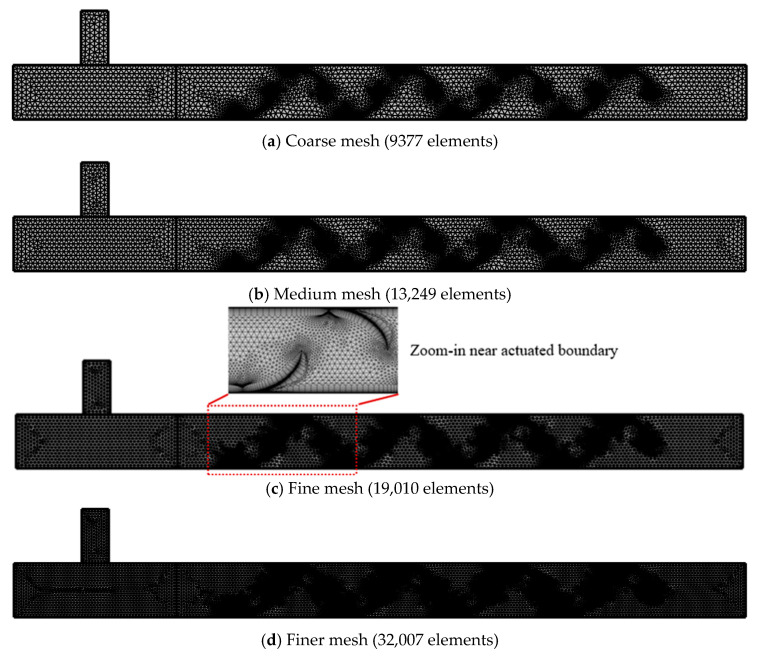
The computational meshes used in the grid-independence study, together with a local zoom-in showing the near-wall refinement around the actuated boundary.

A mesh independence assessment was conducted to verify that the predicted flow and scalar fields were not artifacts of spatial discretization. Four successively refined grids were examined: Coarse (9377 elements), Medium (13,249), Fine (19,010), and Finer (32,007). All geometric, physical, and numerical settings were held fixed across meshes to isolate grid effects. In particular, the actuation amplitude and frequency were set to $A=1.0\times 10^{-3}\;{\;m\;s}^{-1}$ and f=1 Hz, respectively, and the same boundary conditions, solver options, and time-stepping parameters were used for each case. Representative grid topologies are shown in Figure 2. A local zoom-in of the actuated-wall region is included in Figure 2 to show the near-wall refinement and mesh grading used to maintain adequate resolution during wall deformation.

The mesh quality near the moving walls was controlled as described in the previous section, ensuring comparable near-wall resolution (targeting $y^{+}\leq 1$) on all grids. The primary comparison metric was the area-averaged ethanol concentration evaluated in the downstream outlet window (Zone 3), consistent with the study’s performance measures. The convergence was assessed by tracking the approach of this metric from Coarse to Finer meshes and by examining whether the predicted outlet concentration approached an asymptotic value under mesh refinement.

In addition to the mean outlet concentration, the spatial standard deviation of concentration, σ, was evaluated as a supplementary fluctuation-sensitive indicator, because variance-based mixing measures are more demanding than mean values and are more sensitive to under-resolved scalar interfaces. Specifically, the spatial standard deviation of concentration, σ, was computed over Zone 3, which directly reflects the resolved concentration gradients and is therefore more demanding than a mean value. Coarser meshes tend to smooth steep gradients numerically and can underestimate concentration fluctuations, whereas refined meshes better resolve scalar interfaces and yield a higher and more reliable estimate of *σ*. In the present study, σ increased as the mesh was refined from Coarse and Medium to Fine, and the change from Fine to Finer was comparatively small, indicating that the key fluctuation content relevant to variance-based mixing measures was sufficiently resolved on the selected mesh.

Using the finest grid as the reference solution, the area averaged outlet concentration in the evaluation window (Zone 3) was used to quantify mesh convergence. The relative errors of the Coarse, Medium, and Fine meshes with respect to the reference were within a few percent, and the Fine and Finer predictions were nearly indistinguishable at the plotting scale, indicating that the simulations had entered an asymptotic mesh convergence range. Based on this behavior, the Fine mesh was selected for all reported parametric cases as an accuracy cost compromise, and all subsequent trends discussed in this paper are therefore not artifacts of spatial discretization.

### 2.5. Boundary and Initial Conditions

Boundary conditions were specified to isolate the contribution of wall actuation to transport while avoiding outlet reflections and inlet-induced artifacts. The flow was modeled as laminar and incompressible; no turbulence model was employed. Where the solver required placeholder turbulence values for backflow handling, a turbulence intensity of 1% and a turbulent-to-molecular viscosity ratio of 1 were supplied; these placeholders were inactive under the laminar formulation.

**Figure 3:**
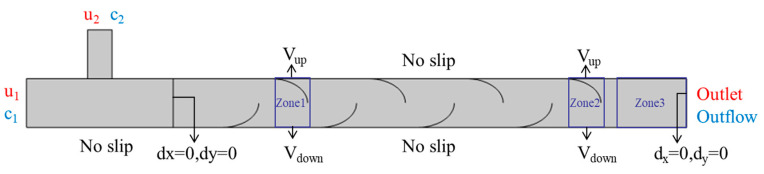
A schematic of the 2D computational domain illustrating the boundary conditions for the coupled fluid flow and mass transport simulation.

As presented in Figure 3, two velocity inlets supplied segregated streams with uniform (“top-hat”) profiles of prescribed magnitudes *u*_1_ and *u*_2_. To define the scalar field, the stream at Inlet 1 consisted of water only with ethanol concentration *c*_1_ (mol m^−3^; commonly *c*_1_ = 0), whereas the co-injected stream at Inlet 2 carried ethanol at a prescribed tracer concentration *c*_2_ (mol m^−3^) in water. Thus, ethanol acted as a passive scalar carried by the liquid phase, and any downstream homogenization arose from the computed flow and diffusion within the device rather than from premixing.

A pressure outlet set to 0 Pa gauge (relative to atmosphere) with backflow suppression permitted the unimpeded egress of disturbances generated by the squeezing motion and minimized spurious re-entrainment at the boundary. For the species equation, a convective outflow with zero normal diffusive flux was imposed so that the composition was determined solely by advection at the boundary; this corresponds to a traction-free condition for momentum and a Neumann condition for the scalar.

All solid boundaries were impermeable to species (zero normal diffusive flux). The stationary walls satisfied the no-slip condition with zero wall velocity. Actuated wall segments in the flexible section enforced the prescribed time-periodic motion via the ALE formulation, with the fluid velocity matching the instantaneous wall velocity to satisfy no-slip on moving boundaries. The walls were assumed to be hydraulically smooth; the roughness parameters were left at solver defaults and were inactive under the laminar model.

Initial conditions: At t = 0, the domain was filled with quiescent water, and the scalar field was consistent with the inlet specification, water only in the Inlet 1 stream and ethanol at concentration *c*_2_ in the Inlet 2 stream, ensuring complete initial segregation at the entry planes.

For clarity in the actuation and performance assessment, the computational domain was partitioned into three regions: Zones 1 and 2 denoted the flexible-wall segments subjected to periodic squeezing, and Zone 3 was a downstream window used to compute mixing metrics (e.g., area-averaged concentration and derived indices). This zoning linked the location of momentum forcing (Zones 1–2) to the region of quantitative evaluation (Zone 3) within a single, self-consistent framework.

In a sensitivity analysis, eight stationary internal surrogate curves were embedded to approximate first-order blockage and streamline deflection associated with ancillary elements; the same boundary conditions described above were applied, and the scalar impermeability of walls was maintained. The case parameters are summarized in Table 1.

For reproducibility, the transient simulations were performed over a total physical time of 30 s with a fixed time step of Δ*t* = 0.01 s. At each time step, the governing equations were iterated until the residuals of all solved variables fell below 1 × 10^−5^, after which the solution was advanced in time. Across the explored actuation frequencies, this time step corresponds to 50, 25, and 20 time steps per actuation period at *f* = 2, 4, and 5 Hz, respectively. A time step sensitivity check confirmed that further reduction in Δ*t* does not produce appreciable changes in key outcome measures such as MI and the sustained-threshold mixing time *t*_s_.

### 2.6. Metrics for Assessing Mixing Homogeneity and Rate

To evaluate mixing performance, four quantitative metrics were employed: the Instantaneous Mixing Index (*MI*, dimensionless), the peak mixing index (*MI*_p_, dimensionless), the Stable-Phase Average Mixing Index (*MI*_avg_, dimensionless), and the Sustained-Threshold Time (*t*_s_, s). All quantities were computed from the simulated concentration field within the designated downstream sampling window (the terminal 25 mm section, hereafter “Zone 3”). *MI*, *MI*_p_, and *MI*_avg_ quantify variance-based homogeneity at different stages of the process, whereas *t*_s_ measures the time required to reach and then maintain a target *MI* level. Variance or intensity-of-segregation-based indices are standard for miscible mixing and enable fair comparisons across geometries and operating conditions; time-to-threshold measures are widely used to characterize transient performance in micromixers and peristaltic mixers [40].

The degree of fluid homogeneity at any time *t* was quantified by *MI*, defined as the complement of a normalized root-mean-square (RMS) deviation of concentration about a reference value *c*_ref_. Normalization uses the RMS deviation under the initially segregated state in the same sampling window. The mathematical expression is as follows (Equation (7)):

(7) $$MI(t)=1-\frac{\sqrt{\frac{1}{N}\sum_{i=1}^{N}{\left(c_{i}(t)-c_{ref}\right)}^{2}}}{c_{ref}}$$

where *c*_i_(*t*) is the concentration at sampling point *i* at time *t*; *c*_ref_ is the well-mixed bulk concentration computed as the flow-weighted average of the inlet concentrations, and *N* is the number of sampling points in Zone 3.

Under the baseline inlet split, *c*_ref_ = 0.9 mol m^−3^ and serves as the target value for complete mixing; for other inlet splits, *c*_ref_ is recomputed accordingly. Values of *MI* approaching 1 indicate a high degree of homogeneity.

The peak homogeneity achieved during the observation window [*t*_0_, *t*_f_] was reported as (Equation (8)).

(8) $$MI_{p}=\underset{t\in [t_{0},t_{f}]}{max}\;MI(t)$$

where [t_0_, t*_f_*] represents the observation time interval.

To account for oscillations in *MI*(*t*) arising from periodic actuation, a stable-phase average was also reported. The Stable-Phase Average Mixing Index was defined as the time average of *MI*(*t*) over a late-time window [*t*_a_, *t*_b_]:

(9) $$MI_{avg}=\frac{1}{t_{b}-t_{a}}\int_{t_{a}}^{t_{b}}\;MI\left(t\right)dt$$

The stable averaging window was set to *t*_a_ = 20 s and *t*_b_ = 25 s. This choice follows a start-up transient of approximately 15 s; by *t* = 20 s, the pressure drop, outlet flow rate, and domain-averaged *MI* had reached repeatable cycle-to-cycle behavior. A 5 s duration spans roughly five actuation periods at the 1 Hz baseline, which is sufficient to smooth intra-cycle oscillations while preserving the underlying dynamics for comparison. Sensitivity checks using shifted or longer windows produced essentially identical values, so this window was adopted for consistency.

As presented in Equation (10), the rate at which a high level of uniformity is attained was characterized by the Sustained-Threshold Time, defined as the first time at which *MI*(t) exceeds a prescribed threshold *θ* and remains above it thereafter:

(10) $$t_{s}=min\left\{t|\forall \tau \geq t,MI(\tau )\geq \theta \right\}$$

Unless otherwise noted, *θ* = 0.9. If *MI*(t) for a given case does not continuously exceed the threshold, *t*_s_ is reported as undefined.

A brief sensitivity check was performed to assess the robustness of the sustained-threshold mixing time definition in Equation (10). The threshold *θ* was modestly perturbed around the nominal value of 0.9, and the sustained requirement was slightly relaxed by adjusting the averaging (or persistence) window. These changes produced only small quantitative differences in the reported mixing times, while the relative ranking of all cases across the explored operating conditions remained unchanged. This confirms that the comparative trends and operating-region conclusions drawn from ts are insensitive to modest choices of *θ* and averaging window settings.

Transient simulations were performed to capture the periodic flexible-wall squeezing. The total simulated time was 30 s, with a fixed time step of Δ*t* = 0.01 s. Within each time step, the governing equations were iterated until the residuals of all solved variables dropped below 1 × 10^−5^, ensuring stepwise convergence. A time-step-independence study confirmed that a further reduction in Δ*t* does not lead to appreciable changes in key outcomes such as the mixing index and the sustained mixing time. Across the investigated actuation frequencies, this time step corresponds to 50, 25, and 20 time steps per period at f = 2, 4, and 5 Hz, respectively.

### 2.7. Experimental Proof-of-Concept and Fluorescence-Imaging-Based Trend Comparison

**Figure 4:**
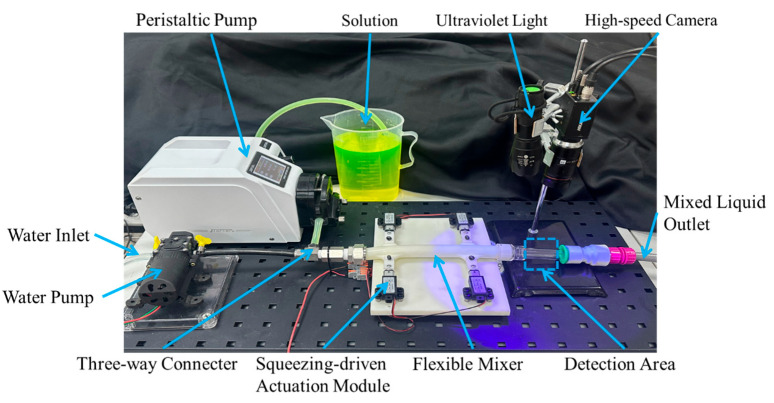
Periodically squeezing-driven mixing platform with fluorescence-imaging measurement system.

Because the physical prototype is a three-dimensional compliant cylindrical device, whereas the CFD framework is a two-dimensional kinematic idealization with prescribed wall motion, the experiment is not used here for direct quantitative validation of the simulated field variables. Instead, it serves as a proof-of-concept and trend-level corroboration platform to test whether stronger and/or faster periodic squeezing improves outlet homogeneity in the same qualitative ordering predicted by the simulations. The comparison therefore focuses on monotonic trends and relative ranking across operating conditions, rather than on one-to-one numerical agreement between experimental and simulated metrics. To experimentally assess the numerically predicted mixing trends, a bench-scale physical mixing platform driven by periodic squeezing was constructed (Figure 4). The working fluids consisted of an aqueous fluorescent-tracer solution and tap water, supplied separately and merged through a three-way connector upstream of the flexible mixer. The water stream was delivered by a DC diaphragm self-priming pump (HY-521, 12 V, maximum flow rate 3.5 L/min; Taizhou Luqiao Huyue Sprayer Factory, Taizhou, China), while the fluorescent solution was delivered by a speed-controlled peristaltic pump (Jieheng BT-600CA drive with a 153Yx pump head, single-channel configuration; Chongqing Jieheng Peristaltic Pumps Co., Ltd., Chongqing, China). The flexible mixer was mounted on a squeezing-driven actuation module and periodically compressed by a micro electric-cylinder actuator (HY05, stroke 10 mm, maximum speed 30 mm s^−1^; generic Chinese OEM manufacturer, Ningbo, China), enabling the controlled deformation of the flexible wall to emulate the prescribed periodic boundary motion in the simulations. To match the simulation intent, the two inlet flow rates were held constant in each run to isolate the effect of actuation parameters on mixing performance. Four representative squeezing conditions were tested: no squeezing, 1 Hz with a working speed of 10 mm s^−1^, 2 Hz with a working speed of 10 mm s^−1^, and 2 Hz with a working speed of 30 mm s^−1^.

Mixing uniformity was evaluated in a transparent detection tube positioned immediately downstream of the mixer outlet. Fluorescence imaging was implemented using a Hikvision machine-vision platform comprising a high-speed industrial camera (MV-CH100-60GC, HIKROBOT, Hangzhou, China) and a fixed-focal machine-vision lens (MVL-KF2528M-12MPE, HIKROBOT, Hangzhou, China). The fluorescent tracer was excited by a UV light source (40 W, 365 nm). During image acquisition, the UV source, camera, and detection region were enclosed with blackout shielding to minimize ambient-light interference. The camera was fixed normal to the detection tube at a working distance of approximately 10 cm, and the optical settings were kept constant across all tests (exposure time 5000 microseconds, gain 23 dB, and frame rate 9 to 11 fps). The imaging location was set about 2 cm downstream of the mixer outlet, and all frames were recorded continuously over the mixing transient and subsequent quasi-steady phase for each squeezing condition.

The recorded fluorescence images were processed using a fixed ROI in the detection tube, and the background intensity was subtracted to correct for ambient light leakage and camera offset. Under dilute-tracer conditions, the measured fluorescence intensity was treated as proportional to the local tracer concentration, enabling an intensity-based evaluation of the scalar homogeneity. The mixing uniformity was quantified using the CV of pixel intensity within the ROI, defined as the standard deviation divided by the mean intensity. For each operating condition, the time history of CV was tracked to characterize transient mixing and stabilization. The mixing process was considered to have reached a stable state when CV remained within plus or minus 2% of its mean value over a continuous time window, indicating statistically stationary behavior under periodic squeezing.

To connect experimental actuation settings with the CFD parameterization, the experimental inputs are reported as the actuation frequency and the programmed peak translation speed of the actuator, which serves as a practical proxy for the deformation rate imposed on the flexible wall. In the CFD model, the corresponding actuation intensity is represented by the prescribed wall-normal velocity amplitude A and the frequency f, enabling consistent trend-level comparisons between experiment and simulation even when absolute magnitudes differ.

The mixing uniformity was quantified from the recorded fluorescence images using the coefficient of variation (CV) of pixel intensity within a fixed region of interest (ROI) in the detection tube. Under dilute-tracer conditions, the measured fluorescence intensity was treated as proportional to local tracer concentration, enabling the intensity-based assessment of scalar homogeneity. The CV was computed as

(11) $$CV=\frac{\sqrt{\frac{1}{n}\sum_{i=1}^{n}(x_{i}-\bar{x}{)}^{2}}}{\bar{x}}$$

where $x_{i}$ is the fluorescence intensity of pixel i within the ROI, $\bar{x}$ is the mean intensity over the ROI, and n is the number of pixels. A larger CV indicates stronger spatial segregation and poorer mixing, whereas a smaller CV corresponds to a more uniform intensity field and improved mixing.

The fluorescence images were processed using a fixed ROI located downstream of the mixer outlet. For each frame, a background image (no fluorescence signal) was subtracted to correct for ambient illumination and camera offset. Under the dilute-tracer condition used in this study, the measured fluorescence intensity was proportional to the local tracer concentration, allowing intensity statistics within the ROI to be used as a proxy for concentration uniformity. The CV was computed as the standard deviation of pixel intensity within the ROI divided by the mean intensity. The resulting time-resolved CV(*t*) curves were used to quantify transient and quasi-steady mixing behavior and to compare trends across operating conditions.

For each operating condition, the time history of CV was tracked to characterize transient mixing and stabilization. The mixing process was considered to have reached a stable state when CV remained within ±2% of its mean value over a continuous time window, indicating that the intensity field had become statistically stationary under periodic squeezing.

To connect the experimental and numerical metrics, it was noted that both the experimental CV and the numerical MI are variance-based indicators of mixing homogeneity. The CV was computed from fluorescence images as the normalized standard deviation of pixel intensity within a fixed outlet ROI, whereas the MI was computed in the CFD framework from a concentration-variance definition normalized to the initially segregated state. Because of these differences in definition, sampling location, and measurement modality, their absolute values were not expected to be numerically identical. However, both metrics reflect the same physical process of variance decay during mixing and therefore should exhibit consistent monotonic trends with time and with increasing actuation intensity (amplitude/speed proxy) or frequency. Accordingly, comparing monotonic trends and relative ranking in CV and MI provides a meaningful basis for qualitative trend comparison between the experiment and the CFD predictions.

To facilitate the comparison between CFD and experiments, the actuation inputs were aligned at the protocol level. In the simulations, the flexible-wall motion was imposed as a sinusoidal wall-normal velocity boundary condition (see Equations (5) and (6)), where A is the peak wall-normal velocity amplitude and f is the actuation frequency. In the experiments, the flexible wall was driven by a linear actuator in periodic reciprocation, and the reported programmed peak translation speed $V_{prog}$ (e.g., 10 mm/s, 30 mm/s) served as a practical proxy for the imposed deformation intensity. Because the actual wall-normal deformation speed depended on compliant wall mechanics and the specific contact/constraint configuration, a strict one-to-one numerical equivalence between $V_{prog}$ and the CFD amplitude A was not assumed. Therefore, the CFD–experiment comparison in this work was conducted at the trend/ordering level: the actuation frequency f was matched between the experiment and the simulation, and increasing $V_{prog}$ in the experiment was interpreted as increasing actuation intensity, analogous to increasing A in CFD.

## 3. Results and Discussion

### 3.1. Mesh Independence Verification

**Figure 5:**
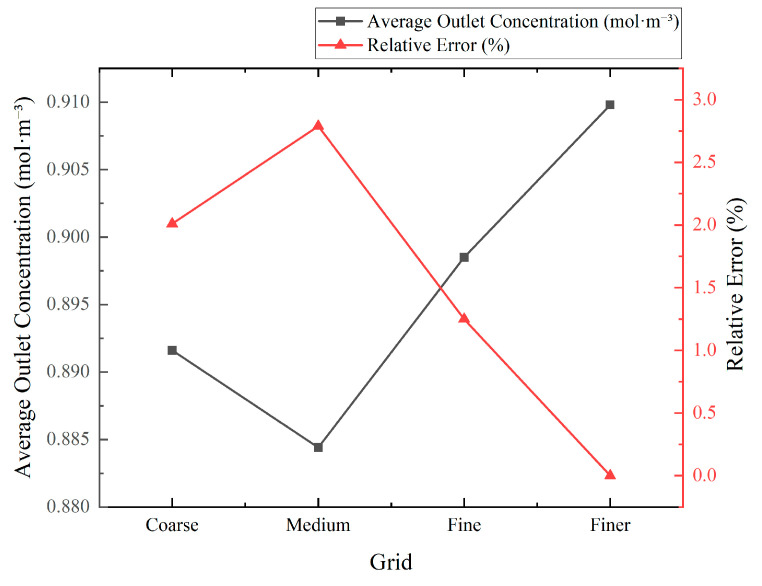
Mesh convergence assessment based on the area-averaged outlet concentration in Zone 3 and its relative deviation from the finest tested mesh.

A mesh independence study was performed using four systematically refined grids (Coarse, Medium, Fine, and Finer) to verify that the outlet concentration is insensitive to spatial discretization. The densest mesh (Finer) was adopted as the reference benchmark, yielding an average outlet concentration of 0.9098 mol·m^−3^. Relative errors were computed as $\varepsilon_{r}=|C-C_{ref}|/C_{ref}\times 100\%$. As shown in Figure 5, the predicted average concentration approaches an asymptote with refinement, and the Fine and Finer predictions are nearly indistinguishable at the plotting scale. Using the Finer solution as $C_{ref}$, the relative errors for the Coarse, Medium, and Fine grids were 2.01%, 2.79%, and 1.25%, respectively. Although the error was not strictly monotonic from Coarse to Medium, the decrease from Medium to Fine and the visual overlap of the Fine and Finer results indicate entry into the asymptotic range of mesh convergence. Based on this convergence behavior, the Fine mesh (19,010 elements) was selected for all reported simulations. It delivered mesh-independent predictions at a substantially lower computational cost than the Finer grid, with only a 1.25% difference from the benchmark in the key metric (average outlet concentration). The trend in Figure 5, moderate variability on the coarser meshes followed by rapid stabilization from Fine to Finer, supports this choice and confirms that further refinement would have a negligible effect on the reported quantities of interest.

The mesh quality was consistently acceptable across all grids and throughout the ALE motion. All meshes satisfied standard thresholds: maximum skewness ≤ 0.30, minimum orthogonal quality ≥ 0.70, and no negative-volume or inverted cells. Aspect ratios in the core region were typically < 10, while larger near-wall ratios were expected owing to the $y^{+}\leq 1$ requirement; these were controlled with a smooth layer growth ratio of 1.15. During the imposed periodic squeezing, dynamic mesh smoothing preserved these quality bounds throughout the cycle, and no remeshing was required.

Because the quality metrics were essentially identical among the tested grids, the small deviations relative to the benchmark were attributed to spatial resolution rather than degradation in the cell quality. The quantitative errors reported above and the qualitative trends this figure justify the adoption of the Fine mesh as the optimal accuracy–cost compromise for the simulations presented in this work.

Table 2 reports the spatial standard deviation of concentration, σ, in Zone 3 as a supplementary fluctuation-sensitive indicator. This quantity is more demanding than the mean outlet concentration because it depends directly on how well sharp scalar gradients and interfacial structures are resolved. σ is not monotonic between the two coarsest meshes, which is consistent with under-resolved scalar interfaces and numerical smearing effects that can bias fluctuation estimates when the mesh is insufficient. Once the scalar gradients are adequately resolved, the estimated fluctuation level stabilizes, and the relative difference between the Fine and Finer meshes is about 6.3%. This level of change is small compared with the actuation-induced differences discussed in the Results, supporting the conclusion that the reported trends in variance-based mixing metrics, including MI and the sustained-threshold mixing time ts, are not artifacts of spatial discretization. Accordingly, the Fine mesh was adopted for the full parametric study as an accuracy–cost compromise.

### 3.2. Effect of Squeezing Actuation on Mixing Efficiency

**Figure 6:**
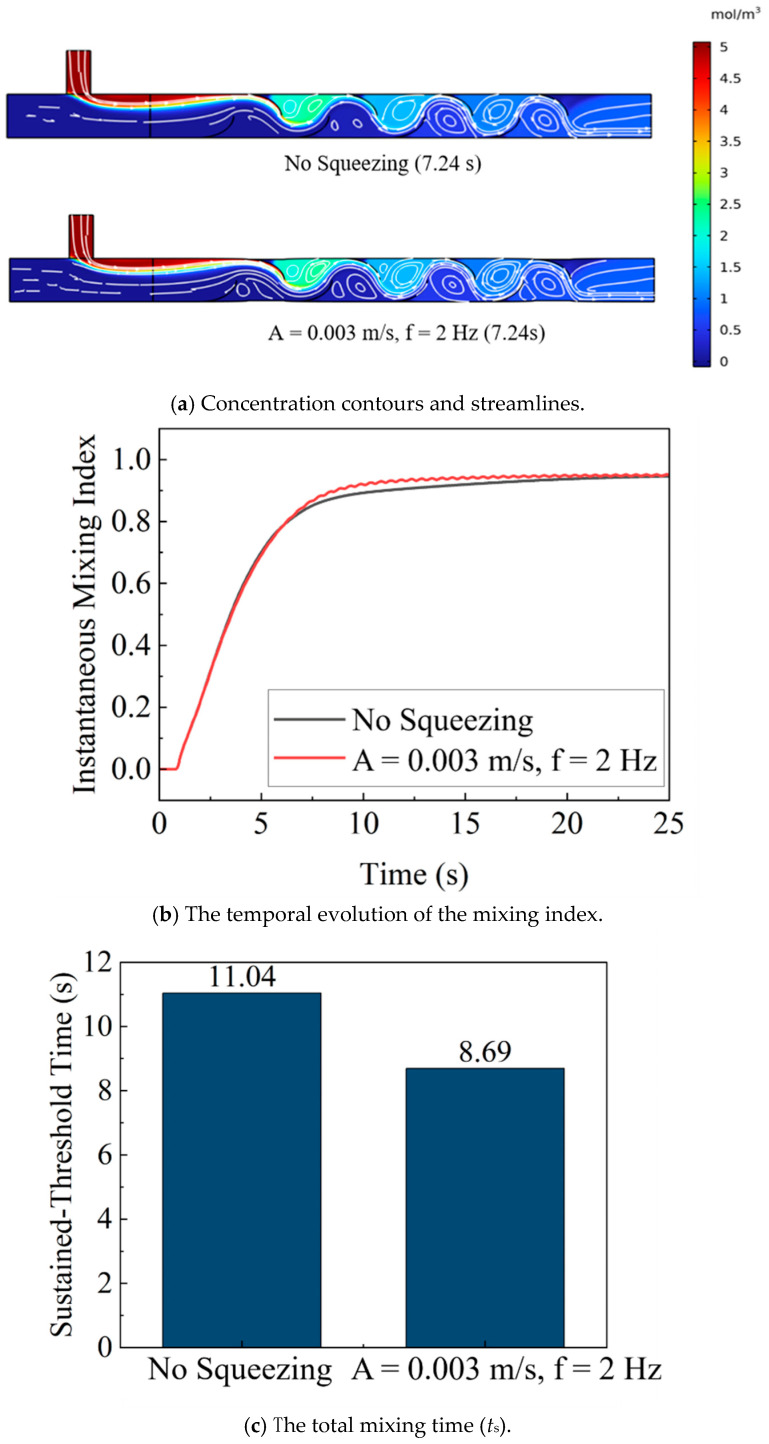
A comparison of mixing performance between the non-actuated case and the actuated case.

All comparisons between the actuated and non-actuated cases in Figure 6 were performed under identical baseline flow conditions. The inlet velocities and total volumetric flow rate were kept the same, and the Reynolds number based on the mean bulk velocity and characteristic hydraulic length scale was matched, so that the observed differences in mixing metrics can be attributed to wall actuation rather than to flow-rate variations. The quantitative impact of squeezing actuation on mixing efficiency was demonstrated by the temporal evolution of the MI. To facilitate a detailed analysis, the mixing process was divided into three distinct phases.

In the early phase (0–5 s), mixing was primarily dominated by the advection of the initially segregated fluid streams. At this stage, the wall motion had not yet accumulated sufficient strain to appreciably stretch or fold the interfaces between the streams. This start-up delay before rapid homogenization was consistent with observations in laminar micromixers and peristaltic or oscillatory devices, where several cycles of deformation were required to build transverse transport [35,41].

In the middle phase (5–10 s), the actuated case showed a markedly steeper increase in MI than the non-actuated case, indicating that periodic squeezing enhanced interface stretching and accelerated scalar homogenization [35,41,42]. In the late phase (t > 10 s), both cases approached a plateau, but the actuated case maintained a higher final MI, demonstrating not only faster convergence to homogeneity but also a better final mixing state.

To investigate the specific impact of wall actuation on mixing efficiency, a baseline case without wall motion was compared to a case with periodic squeezing (amplitude *A* = 0.003 m/s, frequency *f* = 2 Hz). The chosen squeezing amplitude was within a reasonable deformation range for the physical mixer. Figure 6a presents a comparison of concentration contours and streamlines for both the non-actuated and actuated cases at *t* = 7.24 s. In both cases, the internal blade structures generated regular vortices, but the streamlines in the non-actuated case remained relatively smooth within these vortices. The interface between the high-concentration and low-concentration fluids remained distinct, indicating that mixing was limited to convection and diffusion alone. In contrast, when squeezing was introduced, the flow field was significantly perturbed. The streamlines within the vortices became denser and more convoluted, indicating intensified local shear forces and more vigorous vortex dynamics. This enhanced unsteady vortical advection led to greater interfacial stretching and improved mixing.

The enhancement in mixing efficiency was further quantified by the mixing time, *t*_s_, shown in Figure 6b,c. The data clearly demonstrate that actuation significantly reduced the time required to achieve a high level of homogeneity. For the non-actuated case, the mixing time was 11.04 s. However, with periodic squeezing, the mixing time was reduced to 8.69 s, representing a 21.3% reduction. For the representative comparison, the baseline case yielded $t_{s,0}=11.04\;s$, whereas periodic squeezing gave $t_{s}=8.69$ s, corresponding to a reduction of (11.04−8.69)/11.04=0.213, that is, 21.3%. In addition to the reported magnitude, the difference in sustained-threshold mixing time between the non-actuated and actuated cases was assessed using a non-parametric Kruskal–Wallis test across multiple sampled time windows and actuation cycles, and the reduction was found to be statistically significant (*p* < 0.05). This supports that the observed 21.3% decrease reflects a robust effect rather than a sampling artifact. This result provides quantitative evidence that periodic squeezing of the flexible pipe enhanced fluid perturbation and interfacial renewal, thereby accelerating the transport and mixing of the components. These findings confirmed the positive impact of this bio-inspired actuation strategy on overall mixing efficiency. Representative pressure-drop values were extracted from the CFD simulations to complement the design assessment. For the non-actuated baseline (no squeezing), the mean pressure drop across the mixer was Δp ≈ 1804 Pa, whereas under periodic squeezing at *A* = 0.004 m/s and *f* = 2 Hz, the mean pressure drop increased slightly to Δp ≈ 1876 Pa. This corresponds to an increase of less than 4%, indicating that substantial mixing enhancement is achieved with only a modest pressure-drop penalty under the present operating conditions.

### 3.3. Effect of Squeezing Amplitude on Mixing Efficiency

**Figure 7:**
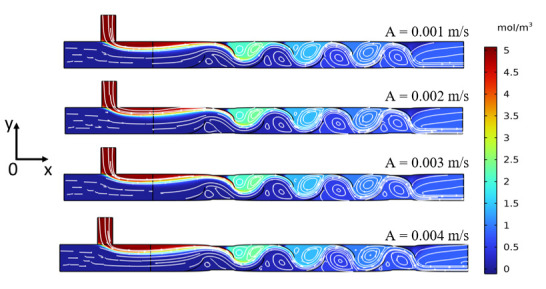
Qualitative effect of squeezing amplitude on mixing performance.

To investigate the influence of squeezing velocity amplitude on mixing efficiency, a parametric study was conducted, examining four different amplitudes (*A* = 0.001, 0.002, 0.003, and 0.004 m/s), with other parameters, such as frequency, held constant. Figure 7 shows the concentration contours and hydrodynamic streamlines for each amplitude at a constant squeezing frequency of *f* = 2 Hz. The results reveal a clear trend that as the squeezing amplitude increases, the deformation of the channel walls in the actuated regions (Zone 1 and Zone 2) becomes more pronounced. This increased wall motion results in more convoluted streamlines, indicating stronger local shear and perturbation. This, in turn, visually correlates with enhanced mixing efficiency and a more homogeneous distribution of the solute.

**Figure 8:**
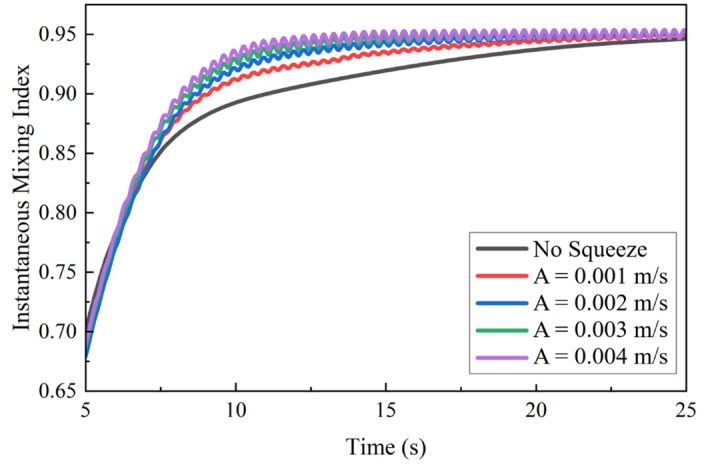
Quantitative effect of squeezing amplitude on the mixing rate.

For a more quantitative analysis, the temporal evolution of the *MI* was plotted for the different amplitudes at a representative frequency of *f* = 2 Hz (Figure 8). In the transient mixing phase (*t* > 5 s), the data show that larger amplitudes (*A* = 0.003 and 0.004 m/s) lead to a more rapid increase in *MI*, reflecting faster mixing progression. A characteristic feature of all actuated cases is the presence of small, high-frequency oscillations in the *MI* curves, corresponding to the system’s response to periodic boundary motion. Over time, all curves gradually approach a stable asymptotic plateau, indicating that each case ultimately achieves a high degree of homogeneity.

**Figure 9:**
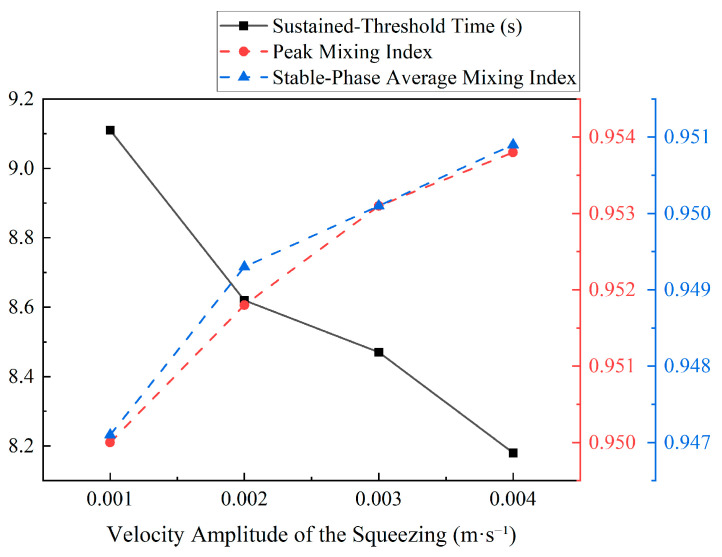
Quantitative analysis of mixing performance metrics as a function of squeezing amplitude.

Figure 9 presents a detailed analysis of the key performance metrics as a function of amplitude. In both the *MI*_p_ and the Stable-Phase, the *t*_s_ is significantly reduced as amplitude increases. The reduction in mixing time, from 9.15 s at A = 0.001 m/s to 8.22 s at A = 0.004 m/s, corresponds to a 10.2% decrease in the time required to achieve a stable, mixed state. This indicated that, while higher amplitudes lead to only marginal improvements in the final homogeneity, they obviously enhance the rate of mixing.

To validate the significance of these differences, a Kruskal–Wallis H test was performed, confirming a statistically significant difference among the *MI* distributions of the different amplitude groups (H = 1586.164, *p* < 0.001). Further analysis through Dunn’s multiple comparisons test revealed highly significant differences between all paired amplitude groups (*p* < 0.001). This statistical analysis provides evidence that increasing squeezing amplitude had a distinct and measurable positive impact on mixing performance, particularly in terms of enhancing mixing rates.

**Figure 10:**
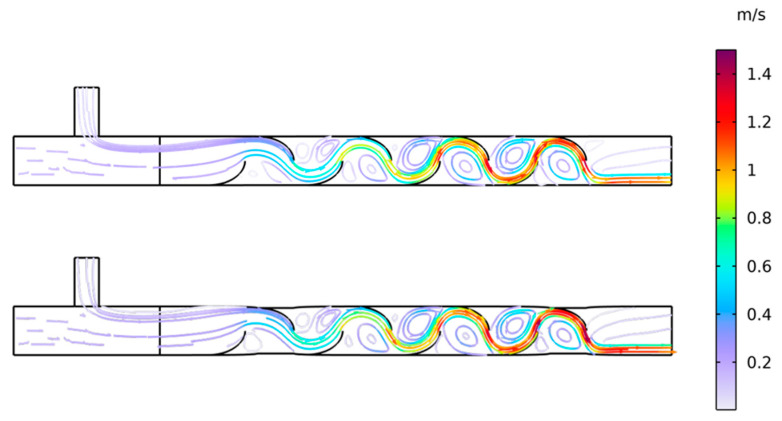
Streamlines colored by velocity magnitude at two representative phases within one squeezing cycle for *A* = 0.004 m/s and *f* = 2 Hz. Color indicates the local velocity magnitude as shown by the color bar.

Figure 10 provides a qualitative visualization of the flow field during periodic flexible-wall squeezing. Comparing the two phases within a single actuation cycle, the streamline patterns show the emergence and reorganization of vortical structures in the squeezing section, together with pronounced spatial variation in velocity magnitude. This unsteady vortical advection enhances interface stretching and folding between the initially segregated streams, thereby increasing interfacial area and accelerating diffusive homogenization under laminar conditions. The repeated alternation between compression and release therefore offers an intuitive mechanistic explanation for the improved mixing metrics observed in the parametric results.

The deformation-driven transport mechanism is supported not only by the monotonic trends in the variance-based mixing metrics, but also by the phase-resolved flow visualization over one squeezing cycle. The streamline patterns at the representative phases of maximum compression and maximum release show the repeated formation, advection, and reorganization of vortical structures in the squeezing section, together with pronounced velocity gradients near the moving wall. These features provide a flow-based explanation for the observed time evolution of *MI*, namely that periodic wall motion promotes repeated interface stretching and folding, thereby increasing interfacial area and accelerating diffusive homogenization under laminar conditions. A fully quantitative diagnostic of this mechanism using vorticity magnitude fields, strain-rate distributions, or Lagrangian particle tracking would provide additional detail on local stretching rates and residence time dispersion, but such analyses are outside the present scope. The current cycle-resolved visualization and metric trends are therefore used to establish a mechanistic, trend-level interpretation suitable for operating-region guidance, while more detailed flow diagnostics are identified as targeted future work.

### 3.4. Effect of Squeezing Frequency on Mixing Efficiency

**Figure 11:**
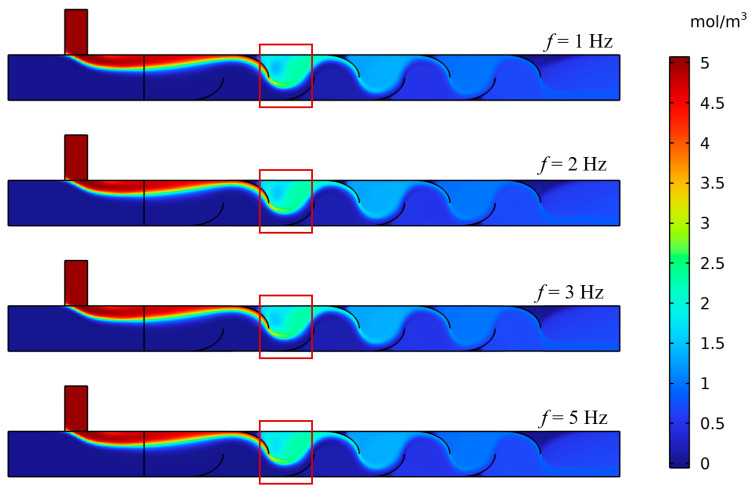
The qualitative effect of squeezing frequency on mixing performance at a constant amplitude.

To complement the analysis of squeezing amplitude, the effects of squeezing frequency on mixing efficiency were examined for four cases (*f* = 1, 2, 3, and 5 Hz) at a fixed amplitude of *A* = 0.003 m/s. For clarity, and to avoid biases from unequal actuation cycles at a fixed dimensional time, concentration contours were reported at two reference instants: a phase-synchronized time corresponding to the same number of cycles for all cases (*N* = 5 cycles, i.e., *t* = 5/*f*), and each case’s *t*_s_, when the *MI* first reaches and remains ≥0.90. The earlier choice of a fixed *t* = 5 s was replaced, as *t*_s_ was roughly 8 s at baseline, and using a fixed time would imply unequal cycle counts across frequencies (e.g., 5 s equals 25 cycles at 5 Hz but only 5 cycles at 1 Hz), which could overstate early mixing for high-frequency cases. By normalizing across frequencies, higher frequencies still show visibly more uniform concentration fields after the same number of cycles and achieve the *MI* threshold in a shorter dimensional time. This normalization aligns with the quantitative trends in mixing time and the statistical tests presented. Thus, Figure 11 illustrates the mixing mechanism after an equal deformation history (*N* = 5), with *t*_s_ providing a fair, outcome-based benchmark across frequencies.

**Figure 12:**
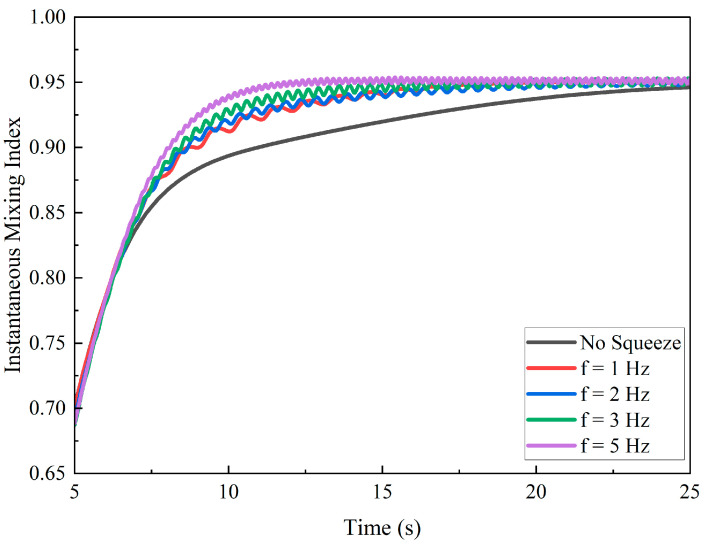
The quantitative effect of squeezing frequency on the mixing process.

A quantitative analysis based on the calculated mixing metrics confirmed and expanded upon these visual observations. The temporal evolution of the *MI*, shown in Figure 12, revealed that higher frequencies result in both a faster rate of mixing and a higher final level of homogeneity. The curves for *f* = 3 Hz and 5 Hz rise more steeply and settle at a higher plateau compared to the lower frequency cases. Figure 12 summarizes these trends, with both the *MI*_p_ and the Stable-Phase *MI*_s_ showing a steady increase with frequency, confirming that higher frequencies improve the ultimate uniformity of the mixture. Concurrently, the *t*_s_ exhibits a general downward trend, indicating a faster mixing process. Specifically, the mixing time decreased from 8.68 s at 1 Hz to 8.10 s at 5 Hz, corresponding to a 0.58 s (6.7%) reduction in the time required to reach a sustained mixed state.

To validate these findings, *MI* distributions sampled over the stable window (*t*_a_ = 20 s to *t*_b_ = 25 s) were compared across frequency groups using a Kruskal–Wallis test with Dunn–Holm pairwise contrasts (*α* = 0.05). Most comparisons were highly significant (e.g., 1 Hz vs. 5 Hz, *p* = 7.41 × 10^−87^), confirming a strong frequency effect. In contrast, adjacent low-frequency groups were not significant after correction (e.g., 2 Hz vs. 1 Hz, *p* = 0.104) and showed a negligible effect size (Cliff’s *δ* = 0.1). This agrees with Figure 11, which exhibits a slight, non-monotonic bump in mixing time at 2 Hz within overlapping confidence intervals.

Physically, the 1–2 Hz range lies in a weak-shear regime, where squeezing-induced transverse transport was comparable to mean convection. In this regime, small frequency changes lead to minimal additional interface stretching. Once the frequency exceeds 2 Hz, the oscillatory Reynolds number increases, and periodic shear becomes dominant, producing the observed monotonic decrease in mixing time and higher *MI* plateaus. The 2 Hz deviation is therefore interpreted as statistically non-significant variability within a low-forcing regime, whereas at higher frequencies, the actuation frequency becomes the primary factor controlling mixing performance.

**Figure 13:**
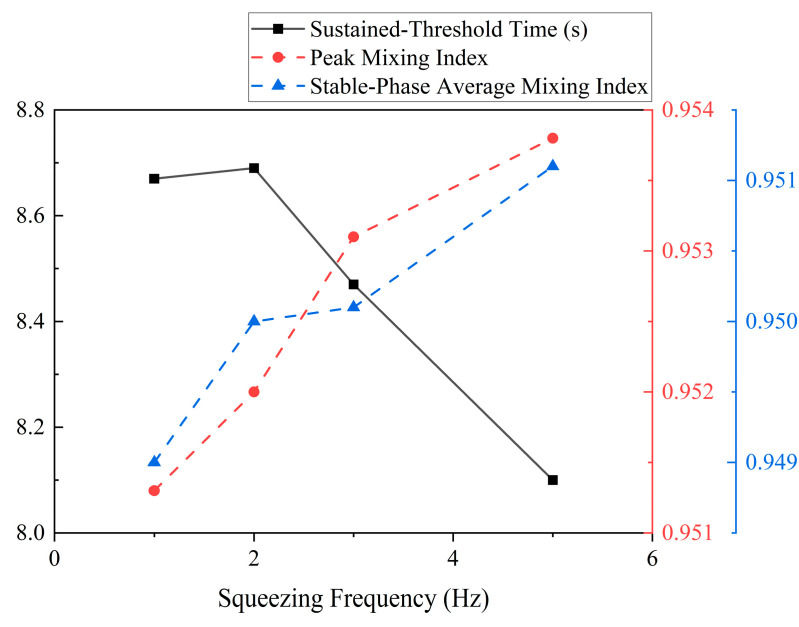
A quantitative analysis of mixing performance metrics as a function of squeezing frequency at a constant amplitude.

Figure 13 shows the temporal evolution of the *MI* for various frequencies, compared to the non-actuated case. The results indicate that increasing the frequency not only accelerates the rate of mixing but also results in a higher final level of homogeneity. Specifically, as the frequency increases, both the *MI*_p_ and the *MI*_s_ exhibit a steady increase, indicating improved mixing efficiency and a higher degree of uniformity in the final mixture. In contrast, the *t*_s_ decreased with higher frequencies, indicating the faster rate at which the system reached a homogeneous state. These findings confirmed that higher frequencies lead to both faster mixing and enhanced homogeneity, which aligned with the quantitative trends observed in the *MI*(*t*) curves, where higher frequencies result in a steeper rise in *MI* and a more rapid approach to a higher plateau.

The phase-resolved flow visualizations support the interpretation that the dominant mixing enhancement arises from deformation-driven transport in the cross-section, manifested as cycle-to-cycle unsteady vortical advection and repeated interface stretching and folding. While three-dimensional effects may exist in the cylindrical prototype, the present results focus on the mixing mechanism directly induced by the imposed wall motion, which is the controllable design input and the primary source of cross-stream deformation in the laminar regime. Accordingly, the conclusions are formulated in terms of operating trends and relative sensitivities to amplitude and frequency, which are expected to remain meaningful even if additional three-dimensional contributions modify absolute values. Extending the analysis to fully three-dimensional simulations with improved geometric fidelity and coupled wall compliance is identified as a next step to quantify any incremental effects from azimuthal and end-wall phenomena.

### 3.5. Response Surface Analysis

**Figure 14:**
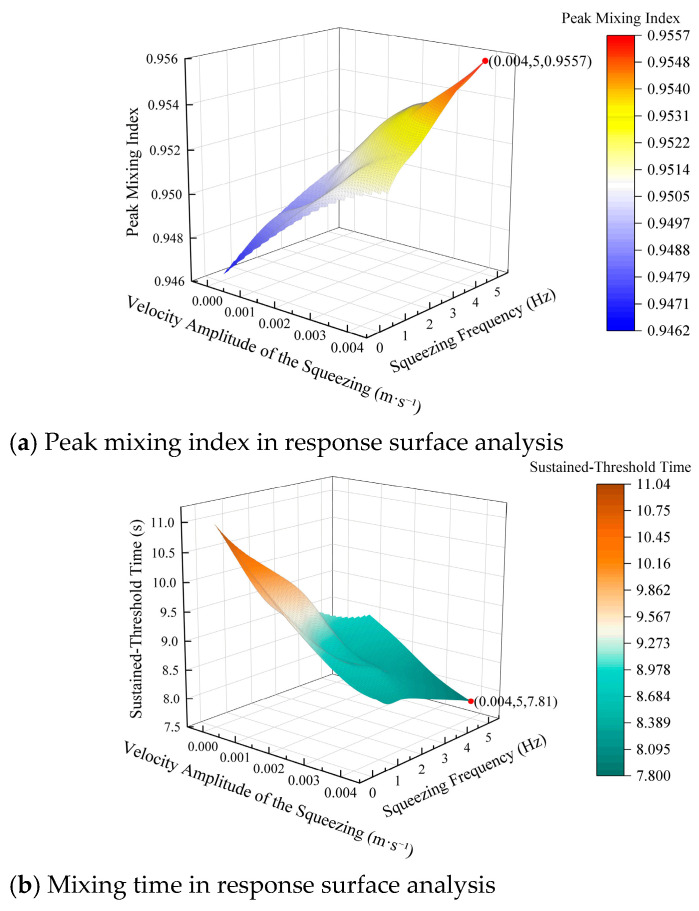
Response surface analysis of mixing performance.

A second-order polynomial surrogate was fitted by least squares to the discrete CFD sampling points to obtain smooth response surfaces for visualization and trend analysis in the explored amplitude–frequency space. The reported goodness-of-fit metrics and residual checks are used only to assess the adequacy of the surrogate representation of the deterministic simulation dataset and should not be interpreted as statistical inference on stochastic variability in the CFD solver output. To comprehensively assess the combined effects of actuation parameters on mixing efficiency, a response surface analysis was conducted. The *A* and *f* were considered as the input variables, while the *MI*_p_ and the *t*_s_ were the key output responses. The response surfaces, presented in Figure 14a,b, model the relationship between these variables and the resulting mixing performance.

Figure 14a illustrates that *MI*_p_ increased monotonically with both amplitude and frequency. This suggests that a higher actuation intensity consistently enhanced mixing homogeneity. The analysis identifies the highest *MI*_p_ value of 0.9557, which occurs at the extreme values of the tested parameters of *A* = 0.004 m/s and *f* = 5 Hz. These optimal conditions achieve the most uniform mixing. These results confirmed that stronger squeezing actuation significantly enhances mixing, aligning with previous studies that highlight the role of increased actuation in improving flow uniformity [41,42].

Figure 14b shows that the *t*_s_ decreases as both amplitude and frequency increase. The minimum mixing time of 7.81 s occurs at the same optimal conditions of *A* = 0.004 m/s and *f* = 5 Hz, indicating that the highest intensity actuation not only improved the final mixing quality but also accelerated the process. This result was consistent with the expected behavior in oscillatory mixers, where higher frequencies and actuation intensities were known to reduce mixing time by enhancing the rate of material stretching and folding [35,42].

The reduced sensitivity at high *A* and *f* reflects the physical saturation of deformation-driven transport under laminar conditions, where finite wall-induced cross-stream deformation and viscous damping limit further increases in interface stretching and variance decay. It is worth noting that this optimum lies near the upper boundary of the explored (*A*, *f*) space. Additional confirmation runs at slightly higher *A* or *f* were not pursued because the 20 mm diameter channel and the actuation hardware impose practical limits on stable wall motion under the present configuration. Importantly, the response trends indicate diminishing returns close to this boundary. For example, at *f* = 5 Hz, increasing A from 0.003 m/s to 0.004 m/s reduces ts only from 8.10 s to 7.81 s and increases *MIp* only from 0.9538 to 0.9557, suggesting that performance is approaching a plateau within the practically accessible range.

The combined interpretation of both response surfaces revealed a clear synergy between squeezing amplitude and frequency. The optimal operating conditions, characterized by high amplitude and high frequency, yield the highest mixing homogeneity (*MI*_p_ = 0.9557) and the fastest mixing time (*t*_s_ = 7.81 s). This confirmed that periodic squeezing, when tuned to high amplitude and frequency, significantly improved both the final quality and speed of mixing. However, from a practical engineering standpoint, while higher-amplitude and higher-frequency actuation results in superior mixing performance in simulations, such intensities are likely to increase energy consumption and accelerate mechanical wear in real-world applications. These factors must be considered when selecting optimal operating conditions for practical systems. The balance between achieving the desired mixing performance and minimizing energy lost or mechanical stress will ultimately define the most efficient operating point. Thus, an optimal operating strategy must account not only for performance metrics but also for economic and engineering constraints, making a trade-off between maximal performance and system longevity.

The response surface analysis indicated that both amplitude and frequency play critical roles in enhancing mixing efficiency. Higher values of both parameters contribute synergistically to faster and more uniform mixing, emphasizing the effectiveness of periodic squeezing as a method for improving mixing performance in bio-inspired systems. However, in real-world applications, the benefits of higher-intensity actuation should be carefully weighed against the increased operational costs and potential mechanical implications.

The diminishing-return behavior observed at higher actuation amplitude and frequency can be explained by the physical limits of deformation-driven transport in laminar flows. Periodic wall squeezing enhances mixing primarily by generating time-dependent shear and unsteady cross-stream advection that repeatedly stretches and folds material interfaces, thereby renewing scalar gradients and accelerating variance decay. As the actuation intensity increases, however, two constraints become increasingly important. First, the effective wall deformation and the associated cross-stream displacement are finite under the present geometry and actuation configuration, so further increases in peak wall speed do not proportionally increase interfacial area or the extent of interface folding. Second, in the low-Reynolds-number regime the flow response is strongly damped by viscosity, and increasing frequency beyond a certain range reduces the time available within each cycle for interfaces to be advected and rearranged before diffusion acts, leading to a progressive saturation in the incremental gain per cycle. Consequently, both *MI* and the sustained-threshold mixing time *t*_s_ exhibit smaller marginal improvements near the upper boundary of the explored (*A*, *f*) space, consistent with the plateau-like trend indicated by the fitted response surfaces.

A second-order polynomial surrogate was fitted by least squares to the discrete CFD sampling points, as summarized in Table 3, to generate smooth response surfaces for visualization and trend analysis in the explored (A, f) space. Because the CFD dataset is deterministic under fixed governing equations, boundary conditions, and numerical settings, the fitted surfaces are not interpreted in a stochastic inferential sense. Instead, the associated uncertainty reflects only the approximation and prediction uncertainty of the surrogate model relative to the sampled design points. 

**Table 3:** Response surface analysis methodology.

Item	Description
Input variables	*A*, *f*
Output responses	*MI*_p_, *t*_s_
CFD sampling points	17 discrete points (covering *A* = 0, 0.001, 0.002, 0.003, 0.004 m/s, *f* = 0, 1, 2, 3, 5 Hz)
Fitted model	$\begin{array}{c} y=\beta_{0}+\beta_{1}A+\beta_{2}f+\beta_{3}(A\cdot f)+\beta_{4}A^{2}+\beta_{5}f^{2} \end{array}$
Fit indicators	$R^{2},$ residual trend and regression coefficient significance (*p*)

### 3.6. Trend-Level Experimental Corroboration and Mechanistic Implications

**Figure 15:**
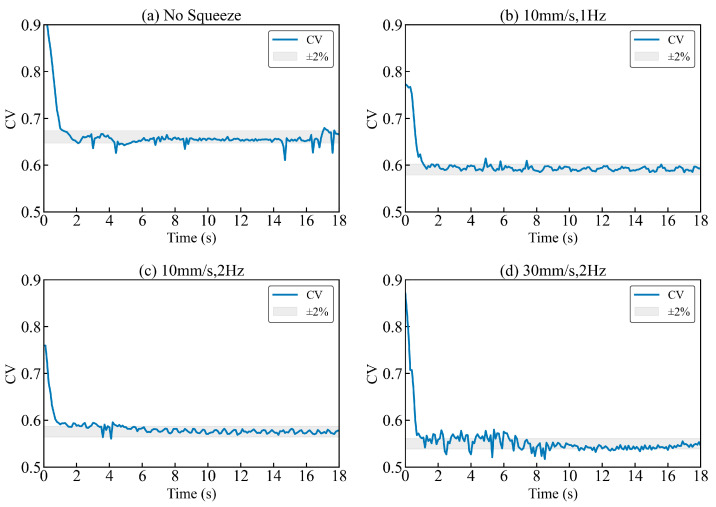
Experimental temporal evolution of outlet fluorescence CV revealing mixing stabilization under periodic squeezing conditions.

Figure 15 shows that, for all cases, the outlet fluorescence CV decays rapidly during the start-up transient and then approaches a statistically stationary plateau (gray band). Under the no-squeeze condition, CV decreases initially but stabilizes at a relatively high level (≈0.65), indicating limited transverse exchange and persistent segregation at the outlet. Once periodic squeezing is applied, the steady CV is reduced monotonically, reaching ≈0.60 at 10 mm s^−1^ and 1 Hz, ≈0.58 at 10 mm s^−1^ and 2 Hz, and the lowest value of ≈0.54 at 30 mm s^−1^ and 2 Hz. Relative to the no-squeeze baseline, the strongest actuation reduces the steady CV by ~17% (0.65 to 0.54), indicating improved outlet homogeneity under stronger and/or faster periodic deformation.

Although the experimental CV and the simulated *MI* are not identical by definition, both are variance-type descriptors of scalar non-uniformity and are therefore appropriate for qualitative trend comparison across actuation conditions. In the CFD framework, mixing performance is quantified using a variance-based MI and a sustained-threshold mixing time (*t*_s_), with an additional stable-phase averaging window introduced specifically to smooth intra-cycle oscillations caused by periodic boundary motion. The experimental CV time histories exhibit an analogous behavior: after a short transient, CV fluctuates around a repeatable plateau under periodic squeezing, consistent with the cycle-to-cycle repeatability implied by the numerical stable-phase concept. Taken together, the decrease in steady CV with increasing squeezing speed and frequency aligns with the simulated observations that stronger actuation enhances final homogeneity and accelerates the approach to a stable mixed state.

Mechanistically, the experimental trends support the CFD-based interpretation that periodic wall deformation promotes mixing by imposing time-dependent shear and repeated perturbations that stretch, fold, and renew material interfaces. Increasing the squeezing frequency effectively increases the number of deformation cycles experienced per unit time, while increasing the squeezing speed (as a proxy for wall-velocity amplitude) strengthens the instantaneous shear and interface deformation per cycle. This combination amplifies interfacial stretching and folding and intensifies local vortical structures, thereby reducing concentration variance more effectively and yielding a lower steady CV. The data also suggest diminishing returns with frequency at fixed speed (0.60 to 0.58 from 1 to 2 Hz at 10 mm s^−1^), whereas increasing speed at fixed frequency produces a clearer gain (0.58 to 0.54 from 10 to 30 mm s^−1^ at 2 Hz), which is consistent with the notion that once cycle-to-cycle renewal is established, additional frequency increments contribute less than strengthening the per-cycle deformation and shear.

Some quantitative discrepancy between experimental CV plateaus and simulated MI levels is expected because the experiment measures a 3D outlet fluorescence field projected through the optical path, whereas the CFD study is based on a 2D idealization with a passive ethanol tracer and a prescribed ALE wall velocity field. Additional factors such as wall material compliance, pump-induced inlet fluctuations, optical non-uniformity, and ROI selection can contribute to residual CV and to small fluctuations around the plateau. Nevertheless, the monotonic improvement with increased actuation strength observed experimentally provides an assessment of the CFD-predicted directionality of performance gains and strengthens the mechanistic linkage between wall motion, unsteady shear, interfacial deformation, and variance decay established in the numerical results.

Each operating condition was repeated in multiple independent experiments. The CV(t) curve shown in the figure corresponds to a representative run selected for clarity. While minor run-to-run differences exist in timing and in instantaneous CV values, all replicates exhibit consistent time-resolved trends. In this study, the experimental measurements are intended primarily to assess the trends predicted by the CFD simulations, rather than to establish an absolute CV value with high-precision uncertainty quantification; the reproducible trends across replicates confirm that increasing actuation amplitude or frequency accelerates homogenization and reduces the final CV level.

Based on the explored parameter space, the actuation amplitude is the primary lever for improving laminar mixing in this device, while frequency plays a secondary but non-negligible role once a sufficient amplitude level is reached. In the low-amplitude regime, increasing frequency alone yields only modest gains because the wall-induced deformation is not strong enough to generate sustained unsteady vortical advection and effective interface stretching throughout the cycle. In contrast, at mid-to-high amplitudes, increasing frequency provides an additional acceleration of homogenization and a small but consistent improvement in the final uniformity, reflecting more frequent cycles of stretching and folding within the same residence time. From a practical design perspective, we therefore recommend selecting the highest experimentally feasible amplitude first, and then tuning frequency to a moderate-to-high range to balance mixing performance and actuation demand. Within the present constraints, this operating region achieves rapid and uniform mixing while incurring only a modest pressure-drop penalty relative to the non-actuated baseline.

From an engineering design perspective, the mixing gains should be interpreted together with the associated hydraulic and actuation costs. Representative pressure-drop values extracted from the CFD indicate that the mean pressure drop increases only modestly under actuation. Specifically, the non-actuated baseline yields Δ*p* ≈ 1804 Pa, whereas periodic squeezing at A = 0.004 m/s and *f* = 2 Hz yields Δ*p* ≈ 1876 Pa, corresponding to an increase below 4 percent. Under fixed flow-rate operation, the required pumping power scales as Phyd = Δp Q, so the incremental hydraulic power penalty is of the same order and remains small relative to the baseline. Actuation-related burden and potential mechanical wear, however, are expected to increase with both peak kinematics and cycle count, which scale with the selected amplitude and frequency and with the total operating time. In the explored range, amplitude is the primary lever for improving mixing, while frequency provides additional benefit mainly once sufficient amplitude is reached and may exhibit diminishing returns near the upper boundary. Therefore, a practical operating choice is to select the highest feasible amplitude first, then tune frequency within a moderate-to-high range to balance mixing performance against actuation demand, rather than increasing frequency unnecessarily once the improvements begin to plateau. Accordingly, the present study establishes mechanistic understanding and trend-level operating guidance for periodic flexible-wall squeezing under laminar conditions, whereas rigorous quantitative prediction for the physical device will require future three-dimensional, compliance-resolved simulations together with denser experimental benchmarking.

## 4. Conclusions

Under laminar conditions, periodic flexible-wall squeezing was shown to enhance mixing by accelerating deformation-driven transport, including interfacial stretching, folding, and scalar-gradient renewal. Within the explored actuation space, stronger wall motion improved both mixing speed and final uniformity, and the best performance was obtained at the high-amplitude/high-frequency end of the tested range. These findings establish the mechanistic basis and operating-region guidance for using bio-inspired squeezing actuation as a low-shear inline mixing strategy.

Across the explored operating space, active wall deformation consistently accelerated homogenization relative to the non-actuated configuration, confirming periodic squeezing as an effective route to enhance mixing at low Reynolds numbers. Quantitative trends showed that, at A = 3 × 10^−3^ m s^−1^ and f = 2 Hz, the required mixing time decreased by 21.3% versus the non-actuated case. At fixed f = 3 Hz, increasing A from 1 × 10^−3^ to 4 × 10^−3^ m s^−1^ shortened the mixing time by 10.2%, while at fixed A = 3 × 10^−3^ m s^−1^, raising f from 1 to 5 Hz reduced the mixing time by 6.6% and improved the final uniformity. Response surface analysis further identified an operating optimum at A = 4 × 10^−3^ m s^−1^ and f = 5 Hz, yielding a peak mixing index of 0.9557 and a minimum sustained-threshold mixing time of 7.81 s.

Mechanistically, the enhancement arises from deformation-driven transport; time-dependent shear, the repeated stretching and folding of material interfaces, and the cycle-to-cycle renewal of scalar gradients collectively intensify variance decay and promote a faster approach to a well-mixed state. These relationships translate into actionable design guidance: increasing actuation intensity improves mixing, but the incremental benefit diminishes at the lowest frequencies, indicating the need for calibrated parameter selection rather than indiscriminate strengthening of excitation.

To substantiate the simulation-predicted trends, a complementary fluorescence-imaging experiment was conducted on a periodic squeezing-driven physical mixing loop, and outlet uniformity was quantified using an image-based coefficient of variation, CV, of fluorescence intensity. Although the experimental CV and the numerical mixing index are defined differently, both are variance-based measures of segregation and therefore support qualitative trend comparison. Experimentally, the stabilized outlet CV decreased monotonically with stronger and faster actuation, dropping from approximately 0.65 under no squeezing to about 0.60 at 1 Hz and 10 mm s^−1^, to about 0.58 at 2 Hz and 10 mm s^−1^, and reaching the lowest value of approximately 0.54 at 2 Hz and 30 mm s^−1^. This monotonic reduction corroborates the core conclusion from the CFD analysis that increasing actuation strength and frequency enhances mixing uniformity and accelerates convergence toward a stable mixed state.

This study demonstrates the feasibility and parametric performance trends of periodic flexible-wall squeezing using an idealized two-dimensional ALE-CFD framework with prescribed wall kinematics, complemented by a fluorescence-imaging experiment intended for qualitative, proof-of-concept and trend-level corroboration of outlet homogeneity trends. Accordingly, the results are positioned to establish mechanistic understanding and operating-region guidance, rather than to provide a fully resolved representation of geometric details and structural compliance. Future work should extend the framework to three-dimensional simulations with improved physical fidelity, including more realistic geometric features and coupled deformation mechanics, and should broaden experimental benchmarking across a wider range of fluids and rheologies, including viscosity variation and formulation-relevant non-Newtonian behavior. These developments are expected to enable more quantitative operating maps, scaling relationships, and control-oriented guidelines for practical deployment. A rigorous assessment of actuation energy requirements and mechanical wear will further require the direct measurements or physics-based modeling of actuator forces, compliance, and friction, which lies beyond the present dataset and is therefore left for future investigation.

## Figures and Tables

**Table 1:** Summary of geometric and operational parameters for the simulation.

Parameter	Symbol	Value
T-junction main-channel length	*L*_1_	60 mm
T-junction inlet-1 branch width	2*R*	20 mm
T-junction inlet-2 branch width	*R*	10 mm
Flexible-section length	*L*_2_	210 mm
Flexible-section inner width	2*R*	20 mm
Inlet-1 bulk velocity	*u*_1_	0.15 m/s
Inlet-2 bulk velocity	*u*_2_	0.06 m/s
Inlet-1 tracer concentration	*c*_1_	0 mol/m^3^
Inlet-2 tracer concentration	*c*_2_	5 mol/m^3^

**Table 2:** Outlet concentration standard deviation (σ) in Zone 3 for different mesh resolutions (mol/m^3^).

Mesh	Outlet Concentration Standard Deviation *σ* (mol/m^3^)
Coarse	0.0357
Medium	0.0323
Fine	0.0624
Finer	0.0666

## Data Availability

The data presented in this study are available on request from the corresponding author.

## Abbreviations

ALE	Arbitrary Lagrangian–Eulerian
CFD	Computational fluid dynamics
MI	Instantaneous Mixing Index
MIp	Peak mixing index
MIavg	Stable-Phase Average Mixing Index
ts	Sustained-Threshold Time
CV	Coefficient of variation
ROI	Region of interest
